# Enabling Secure Data Exchange through the IOTA Tangle for IoT Constrained Devices

**DOI:** 10.3390/s22041384

**Published:** 2022-02-11

**Authors:** Alberto Carelli, Andrea Palmieri, Antonio Vilei, Fabien Castanier, Andrea Vesco

**Affiliations:** 1 Cybersecurity Lab, Connected Systems and Cybersecurity Area, LINKS Foundation, 10138 Turin, Italy; andrea.vesco@linksfoundation.com; 2 System Research and Applications, STMicroelectronics, 73100 Lecce, Italy; andrea.palmieri@st.com (A.P.); antonio.vilei@st.com (A.V.); 3 System Research and Applications, STMicroelectronics, 20010 Cornaredo, Italy; fabien.castanier@st.com

**Keywords:** secure data exchange, IoT, DLT, IOTA Tangle, hardware secure element, cybersecurity

## Abstract

Internet-of-Things (IoT) and sensor technologies have enabled the collection of data in a distributed fashion for analysis and evidence-based decision making. However, security concerns regarding the source, confidentiality and integrity of the data arise. The most common method of protecting data transmission in sensor systems is Transport Layer Security (TLS) or its datagram counterpart (DTLS) today, but exist an alternative option based on Distributed Ledger Technology (DLT) that promise strong security, ease of use and potential for large scale integration of heterogeneous sensor systems. A DLT such as the IOTA Tangle offers great potential to improve sensor data exchange. This paper presents L2Sec, a cryptographic protocol which is able to secure data exchanged over the IOTA Tangle. This protocol is suitable for implementation on constrained devices, such as common IoT devices, leading to greater scalability. The first experimental results evidence the effectiveness of the approach and advocate for the integration of an hardware secure element to improve the overall security of the protocol. The L2Sec source code is released as open source repository on GitHub.

## 1. Introduction

Internet-of-Things (IoT) systems enable the collection of data from an increasing variety of sensors for analysis and evidence-based decision making. Such systems have two main additional requirements today (*i*) the need for (near) real-time performance to serve their function avoiding offline data analysis and (*ii*) end-to-end security with data source authentication, data confidentiality and integrity from sensor to remote site where data is stored and processed. End-to-end security issues are of paramount importance and they often drive the selection of the solution [1,2,3,4].

The common method of protecting data transmission in sensor systems is the Transport Layer Security (TLS) [5] or its datagram counterpart the Datagram Transport Layer Security (DTLS) [6]. (D)TLS is an incredibly powerful and flexible secure protocol to build secure communication channels between devices [7]. A secure channel ensures authentication of one of the entities or mutual-authentication, confidentiality and integrity of the data exchanged. In practice TLS is used to build a point-to-point secure channel between sensor device and the Edge or Cloud device. Any scenario comprising more than one point dedicated to data analysis is deployed with data duplication/exchange at Edge or Cloud level because it is not suitable to open more than one TLS channel from the IoT device. IoT devices are typically resource-constrained and TLS consumes those resources.

Nowadays, Distributed Ledger Technologies (DLTs) are another relevant and viable option to underpin data exchange while leveraging important security features such as data immutability and verifiability [8,9]. When dealing with largely distributed sensor systems producing data with high throughput, the IOTA Tangle [10] in its new Chrysalis’ version is a valuable choice in the Authors’ opinion.

Transactions on the Tangle require no fees and nodes of the network constantly validate transactions at full speed. A transaction is considered valid when it has a reference to a special one called milestone. The Proof-of-Work (PoW) is not meant to be part of the validation process hence to secure the network as in blockchains but only to discourage spam transactions on the Tangle. A node willing to broadcast a new transaction over the network is expected to validate two previous transactions. As a result, the more incoming transactions, the fastest consensus and transaction validation. In principle the overall throughput is infinite, in practice the low-complexity PoW and consensus process define the throughput limit. The working principles of the Tangle, make it scale with the number of incoming transactions hence an option to serve the IoT and sensors’ world. Moreover, The Tangle is designed to offer the capability to store data with a transaction of a special message carrying a payload of type *indexation* [10]. A transaction with an indexation message anchors the data to the Tangle. Any node willing to consume that data retrieve it at the specified *index*. A protocol working at Layer 2 (L2) (i.e., on top of IOTA Layer 1 responsible to interact with the Tangle), is used to structure data over the Tangle and enable (*i*) transmission of complex data streams and (*ii*) simple data retrieval. Such a L2 protocol is a cryptographic protocol that secures data transmission from end-to-end over the Tangle. It provides the primitives to structure a stream of data over the Tangle, to prove data source and data ownership, to cipher and decipher data and to easily retrieve and consume the data stream while verifying it. The Tangle itself provides data immutability and integrity.

The combination of a L2 cryptographic protocol and the Tangle is an alternative means of secure transport to TLS enabling multi-point to multi-point secure data transfer. This combination provides a trust layer for any distributed sensor system to securely exchange data in a (near) real-time fashion. Not only, data are persistently anchored to the Tangle hence they can be verified and consumed at any time also *a posteriori*. For the sake of completeness, data quality and source reputation are other two main point of attention, any existing solution can be applied on top of such a new IOTA secure transport. Addressing these points is out of the scope of this work.

It is worth noting that the Tangle is a possible standard interface for data exchange. Heterogeneous and independent systems, each one designed to fit a specific purpose, can anyway interact with each other without the need to re-design and develop new custom data exchange interfaces. Any data source has only to grant access to its data stream to the other system.

The IOTA Foundation has developed two L2 solutions. The first is the Masked Authenticated Messaging (MAM) [11] and the second is STREAM [12]. The MAM works with a legacy version of the IOTA Tangle that is no longer available and it is developed in Javascript language. STREAM replaces MAM adding new interesting cryptographic features and it is currently under further development to work with the new Chrysalis version of the IOTA Tangle. STREAMS is developed in RUST language. The main problem with these frameworks is that they do not maintain the promise of the IOTA ecosystem to support the IoT and sensors’ world. They are, in fact, most suitable for desktop applications due to the programming languages selected. Moreover, they have not been designed taking into consideration the typical limitations of IoT constraint devices such as computational capacity, memory availability and low-energy consumption requirements. All in all, these solutions cannot be deployed as is in real-world constraint sensor systems.

This paper presents L2Sec, a cryptographic protocol suitable for IoT constraint devices based on microcontrollers developed from scratch in C Language. L2Sec provides all the capabilities for a constraint IoT device to structure a stream of data over the Tangle and enable secure data exchange. L2Sec is a cryptographic protocol to structure, secure and navigate data through the Tangle. Moreover, L2Sec is enhanced to take advantage of an Hardware Secure Element to build an HW root-of-trust at the IoT device and further improve the security of the overall solution with a secure-by-design approach. L2Sec is designed, developed and tested on a STM32L4+ Discovery kit IoT node [13] with the secure element STSAFE A110 [14].

This paper is organised as follows. Section 2 provides an overview of a general IoT system and discusses the target system. Section 3 presents the existing L2 solutions for secure data exchange over the IOTA Tangle. Section 4 presents the L2Sec cryptographic protocol and details its implementation, addressing the security design choices. Section 5 focuses on the integration of an hardware secure element able to provide additional security features to L2Sec. Section 6 presents the experimental results achieved with an implementation of this protocol on real IoT constraint hardware. Finally, Section 7 summarises and concludes the paper providing an overview of the future works.

## 2. System Overview

A common high level architecture of an IoT system comprises a set of constrained devices, a gateway and one or more remote servers. The constrained devices deployed on the field perform limited tasks, i.e., they fetch data coming from different sensors and send it to a remote server through the gateway. The gateways have instead relaxed constraints and are in general more powerful because they need to be able to interface with several others IoT nodes and other gateways of the system. Moreover, they present additional computational power to provide a certain degree of scalability as well as integration capabilities for coexistence with other systems. The gateways use powerful processors optimized for networking, large memories and present no constraints on power supply. Typically, they run an operating system with their own applications and provide the possibility for remote management and monitoring. The remote servers store, process and visualize data collected from the sensors through a plethora of application tools. Their tasks are application-dependent and, in general, the most various. Nowadays, resorting to a cloud platform is a common choice for scalability purposes and for ease of integration with other systems.

**Figure 1:**
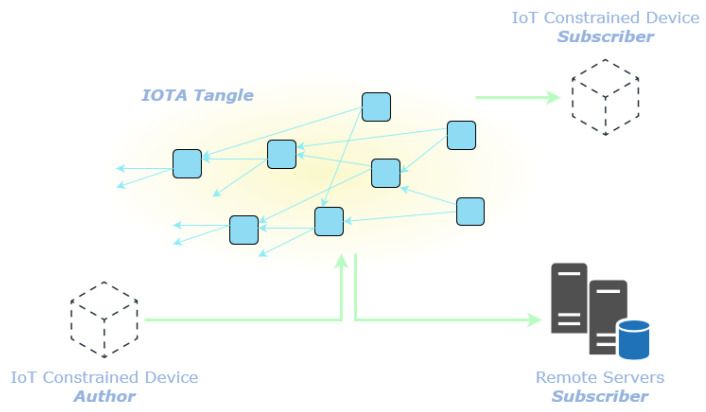
High-level system architecture. The IoT constrained devices act as Authors producing data and transmitting it to the Tangle. The Subscribers are either constrained or not-constrained devices that access the Tangle to retrieve the data.

This work focuses on a slightly different high-level system architecture represented in Figure 1. The IoT constraint devices (i.e., the target devices) communicate with each other and with the servers through the IOTA Tangle [10]. Each IoT constraint device interacts with the IOTA Tangle through an IOTA node [15] acting as the gateway of the distributed ledger.

In this work the target device is a microcontroller-based system equipped with networking capabilities and thus able to communicate with a IOTA gateway through a network peripheral (e.g., WiFi, LoRa, Bluetooth). For the sake of clarity several network hops can exists between the target device and the IOTA gateway. A general overview of the characteristics of the target device is given in Section 2.1, while the IoT system for testing purposes and its physical details are described in Section 6.1.

The IOTA Tangle [10] is considered as a *secure means of transportation* of the data collected by the sensors on the target devices. Other nodes of the network (either constrained or not) can consume the data by retrieving it from the IOTA Tangle. Leveraging the DLT enable point-to-point, point-to-multipoint and multipoint-to-multipoint secure communication among devices and servers deployed on the field and geographically scattered.

### 2.1. Target Devices

The number of nodes of an IoT system is quite high. Therefore, it is important to contain the cost of any single device as low as possible to contain the price of the overall IoT system. For this reason, these devices are simple and their features are limited (e.g., data collection and transmission). These devices are microcontroller-based systems, thus are limited under several domains. In particular, their limitations affect the following domains:

- **Computational capabilities:** lower clock frequencies compared to normal computers limit the throughput of operations. Moreover, the hardware of the target devices is lacking the support of accelerator peripherals (e.g., GPUs);
- **Memory:** the memory size (both ROM/Flash and RAM memories) of microcontrollers is very limited. This factor negatively influences not only the application running, but it might also have an impact on future updates of the firmware;
- **Physical size:** usually the devices are deployed as part of other systems, sometimes requiring also the physical size to be contained (e.g., smaller form factor);
- **Power consumption:** in case the nodes are isolated or too far from power sources, the devices use batteries as a source of energy, leading to shorter time of activity. Moreover, environmental constraints can also dictate limits on emission and power consumption;
- **Networking:** devices are equipped with network peripherals which might offer a limited bandwidth;
- **Costs:** devices require an higher NRE-cost (Non-Recurrent Engineering cost) which can only be spread if the number of produced devices is high. However, each device will account for the cost of the physical materials and components employed, as well as the cost of its deployment on-the-field.

For an additional and more thorough classification of the node constraints the reader can refer to [16]. The limitation domains listed above are inter-dependent, leading also to require a careful and complex design phase of a target device. Although technological progress is helping, in particular on computational capabilities, the cost still represents a limiting factor for the available features equipped on a node.

## 3. Securing Data over the Tangle

The IOTA Foundation developed two L2 protocols to structure and navigate data over the Tangle. These protocols present different cryptographic features, but they also present one common drawback, i.e., they are designed to work on a full-featured system but not on constrained devices.

### 3.1. Masked Authenticated Messaging (MAM)

Masked Authenticated Messaging (MAM) works with the legacy version of the IOTA Tangle. MAM allows any device to publish data in transactions over the Tangle. It can be used by a device to securely anchor a data stream to the Tangle, while only authorized devices are able to read, reconstruct and consume the data stream.

The legacy IOTA Tangle introduced, for the first time, the concept of zero-value transactions to write a data on the Tangle. The IOTA L1 protocol is responsible for transacting the data, but the transaction is not protected or checked for the purpose of verifying the authenticity of the data. MAM is the L2 protocol that provides the way for a device to protect and structure a data steam over the Tangle and for any other devices to decrypt the data stream and verify its authenticity.

The MAM introduced the concept of data channel. Only the owner of a channel can publish data onto a channel and any malicious attempt of an adversary to write fake data over the channel or to take control of the channel can be easily detected by devices authorized to read and consume the data of the channel. MAM channels are therefore a simple way of authenticating that data were published by a given device. As soon as a device publish data to its channel, it receives a channel ID, which is the identifier that allows other device to subscribe to the channel and fetch the data stream. Any data on a channel is accompanied by the information of the next address that contains the next data in the channel.

Public, Private and Restricted channel modes of operations are available. Public channels use the root of a Merkle Tree as the address of the transaction that contains the MAM data, therefore any device, given the channel ID, can decrypt data using the address as the decryption key. Private channels use the hash of the root of a Merkle Tree as the address of the transaction that contains the the MAM data, therefore only the device with the original root can decrypt the data. Finally, the Restricted channels adds an authorization pre-shared symmetric key to private mode. The address of the transaction that contains the MAM data is the hash of the side key and the root of Merkle tree, therefore only the devices with the original root and the pre-shared key can decrypt the data.

The most comprehensive and detailed explanation of MAM working principles can be found at [17] MAM has been developed for legacy version of IOTA protocol, it makes use of ternary coding and quantum-proof cryptography and it is available in Javascript language at [11].

### 3.2. STREAMS

The IOTA Foundation has recently developed STREAMS. It is an organizational tool for structuring and navigating secure data through the Tangle [12]. It is essentially a framework for cryptographic secure messaging applications.

STREAMS allows any device to organize messages (i.e., data) in a uniform and inter-operable structure underpinned by the Tangle that guarantee the integrity and immutability of the data structure. A device, called Publisher, can send messages in a Stream for everyone else and/or restrict access to messages and make it private using public key encryption. Any other devices willing to consume messages, called Subscribers, can subscribe to a Stream and pull information from the Tangle. Subscribers can also contribute messages to a Stream using different types of cross referencing techniques. Thus, Subscribers can also publish unsigned messages, in contrast to MAM where only channel owner could publish data.

Moreover with the new linking techniques each message is linked to another enabling the way to build complex data structures more flexible than linear chain as in MAM. STREAMS also redesigns the message types. STREAMS adds several types making it simple to publish different messages in the same channel, and to distinguish them based on their specific headers.

Finally, STREAMS improves the channel access control, in fact it is possible to apply a different cryptographic mechanism to each message based on its type to make different access control rules.

STREAMS is written in RUST language and it is Available at: [18].

Both MAM and STREAMS provide efficient solutions for structuring secure data over the Tangle. The programming languages employed to develop these protocols are not suitable for constrained IoT devices, which represent the majority of the devices deployed in real-world sensor applications. Moreover, the MAM protocol has been deprecated and replaced by STREAMS.

## 4. L2Sec—A Cryptographic Protocol for Constraint IoT

### 4.1. Design Principles and Features

L2Sec is a lightweight cryptographic protocol for structuring and navigating secure data through the IOTA Chrysalis Tangle. It is designed to be (*i*) sufficiently lightweight to run on constrained IoT devices (i.e., MCU-based platforms also without operating systems), (*ii*) suitable for sensors application data model, such as one single publisher producing time sequenced data, and (*iii*) modular, such that the building block can be easily employed in other applications and extendable to ease the integration of additional features and fields.

L2Sec protocol leverages the specific Chrysalis message payload called *indexation payload* to anchor the data to the Tangle. The indexation payload is composed of an *index* coupled with some arbitrary data (i.e., application data). Any L2Sec protocol message is therefore encapsulated within the indexation payload.

L2Sec structures a data stream as a single-link chain over the Tangle. Every piece of data of the stream is chained to the next one with another index. Any subscriber of the data stream is able to reconstruct it, starting to read at an arbitrary index over the Tangle and following the chain of data linked each other. Essentially, every data message contains the current index and the index of the next message.

L2Sec protocol employs natively the binary encoding proper of IOTA Chrysalis Tangle, thus avoiding the need of additional data conversion to and from trits (i.e., ternary data representation) used by the legacy version of the IOTA Tangle.

Finally, since data is anchored to the Tangle in plain-text, L2Sec adds a security layer composing an Authenticated Encryption with Associated Data (AEAD) [19].

In order to limit the dependencies on external libraries, L2Sec uses the same cryptographic functions used by the IOTA client [20] provided by Sodium library [21].

The design of the L2Sec protocol enables the integration of an hardware secure element to which outsource cryptographic operations. Moreover, the secure element can be employed as hardware Root-of-Trust (RoT) and as source of a unique electronic identity of the IoT device. This paper describes a version of L2Sec employing such HW device in Section 5.

### 4.2. Operating and Security Principles

#### 4.2.1. Payload Structure

**Figure 2:**
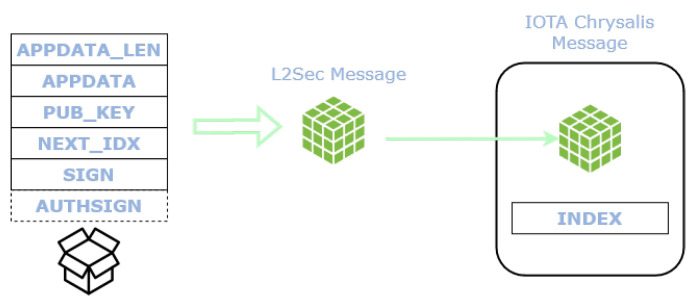
Structure of fields composing an L2Sec message (**left**) and IOTA Chrysalis message (**right**).

A L2Sec message is encapsulated in the indexation payload of a IOTA Chrysalis message. The structure of IOTA Chrysalis message and of L2Sec message is shown in Figure 2.

#### 4.2.2. Message Chaining

**Figure 3:**
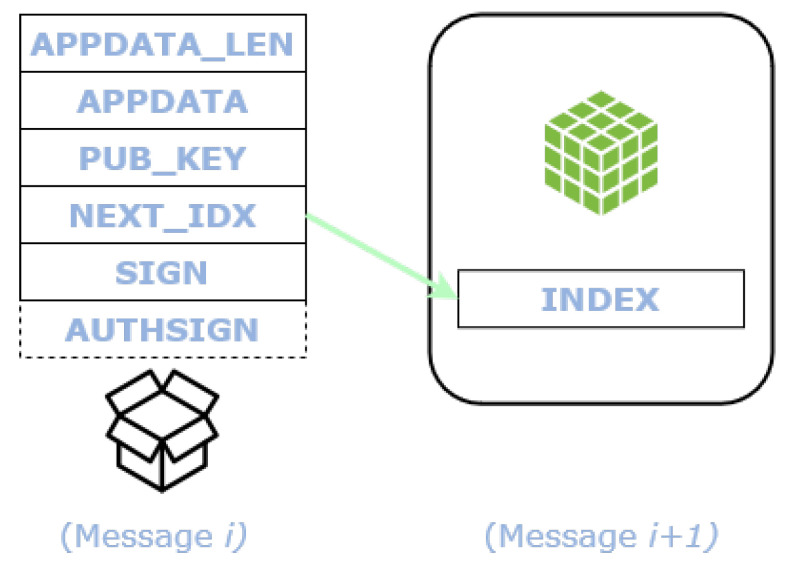
Chaining of L2Sec messages to realize a data stream.

Higher level protocols or applications willing to employ L2Sec, enclose their data in the APPDATA field and its length in APPDATA_LEN field. For data exceeding the maximum data length of a single L2Sec message and for continuous data transmission, a sequence of data has to be linked. The chaining of these messages is realized through the NEXT_IDX field which contains the index of the next message of the stream to look for in the Tangle. Figure 3 show the chaining mechanism.

Realizing the chain through a single link between messages, allows any subscriber to read a data stream in only one direction. This property is intentional, as it inhibits the retrieval of previous information (i.e., past messages belonging to the same data stream).

**Figure 4:**
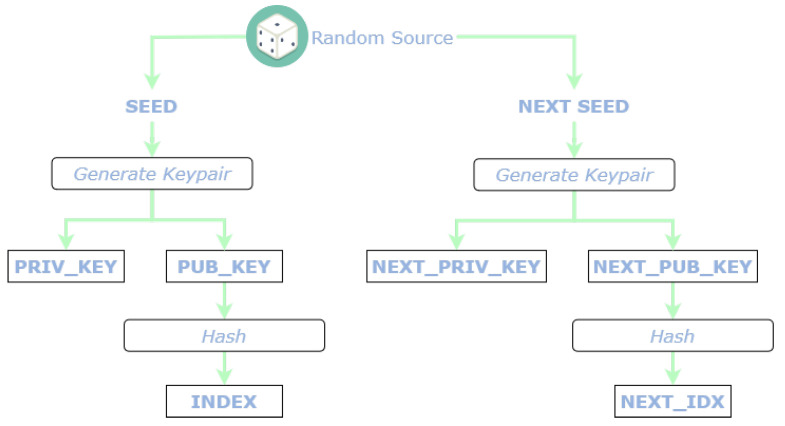
Generation of Index and Next Index fields.

The Figure 4 depicts the flow for the generation of INDEX and NEXT_IDX. L2Sec generates a secret key and a corresponding public key deterministically from a random seed. The key-pair is based on edwards25519 curve. The index of a L2Sec message is then calculated as the result of the hash function of the public key. Moreover, each L2Sec message contains the link to the next index (NEXT_IDX), in order to implement a continuous data stream. The NEXT_IDX is computed in the same manner, starting from a different key-pair.

#### 4.2.3. Data Ownership

Every message contains the field SIGN calculated by signing a digest *h* with the private key (PRIV_KEY) of the key-pair as in Equations (1) and (2).

(1) h=H(APPDATA_LEN+APPDATA+PUB_KEY+NEXT_IDX)

(2) SIGN=signature(h|PRIV_KEY)

**Figure 5:**
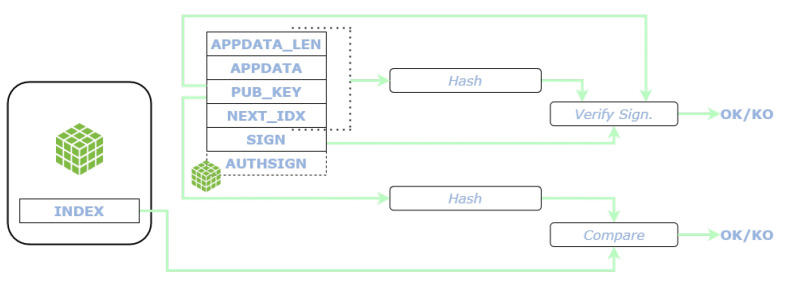
Verifications of an L2Sec message.

A subscriber willing to verify the message recomputes the same hash and verify the signature1 with the public key (PUB_KEY) enclosed in the message itself. Additionally, it verifies the that the hash of the PUB_KEY matches the index of the message retrieved. Figure 5 shows the use of the fields of an L2Sec message for the verification of the data.

The two verifications are performed at the reading of every message. It has to be noted that an adversary who wants to hijack the next message to another malicious stream would need to discover the public key employed to derive the next_index and put it in its message. On the receiver side, the signature and the public key are used to perform verification and to guarantee that no recipient can use the discovered NEXT_IDX to append its chain of messages because it does not know the key-pair used to derive the NEXT_IDX. Basically, this design allows a subscriber to verify that the data is coming from the same source.

L2Sec adopts EdDSA (Edwards-curve Digital Signature Algorithm) over the edwards25519 elliptic curve [22,23] with hashing algorithm BLAKE2b [24] for integrity signature.

#### 4.2.4. Authentication

**Figure 6:**
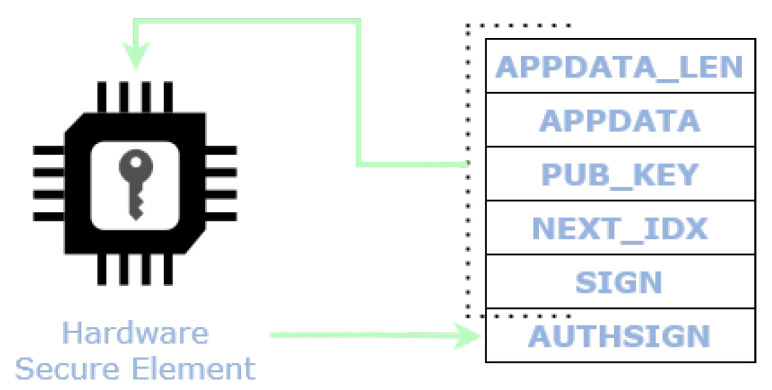
L2Sec message with Authentication Signature generated by a Hardware Secure Element.

The SIGN field does not provide the authentication of the identity of the author, given that the key-pair used is for the sole purpose of implementing the chaining mechanism between messages. To prove and make it possible to verify the identity of the data source, L2Sec requires an additional key-pair associated to the electronic identity of the IoT device. Therefore L2Sec defines an additional field, called *Authentication Signature* (AUTHSIGN), which provides a way to identify and authenticate the source. This signature is calculated by means of the private key and it authenticates all other fields of the L2Sec message. The private key might be stored within an hardware secure element together with its public key certificate. A subscriber that wants to verifies the source of the data must verify this signature by means of the public key of the source, after validation through a trusted Certification Authority. The Figure 6 shows the complete L2Sec message.

The algorithm employed for the authentication signature can be the same used for the integrity signature. Nevertheless, the key-pair used must be different. In the design of L2Sec, the private key is safely and secretly stored into the secure element. Usually, such peripherals expose security primitives realizing cryptographic algorithms. The security primitives supported are strictly dependent on the underlying hardware element and vary on a vendor-model basis. L2Sec protocols employs ECDSA (Elliptic Curve Digital Signature Algorithm) over the NIST Curve P-256 with SHA256 digest for authentication signature. ECDSA is a better alternative to both RSA and DSA for producing digital signatures, and suitable for hardware implementation [25]. ECDSA with a 256-bit key offers the same level of security for the RSA algorithm with a 3072-bit key [26]. Thus, ECDSA can be considered as an optimal alternative, with respect to DSA and RSA, for digital signatures in resource constrained devices.

#### 4.2.5. Encryption

**Figure 7:**
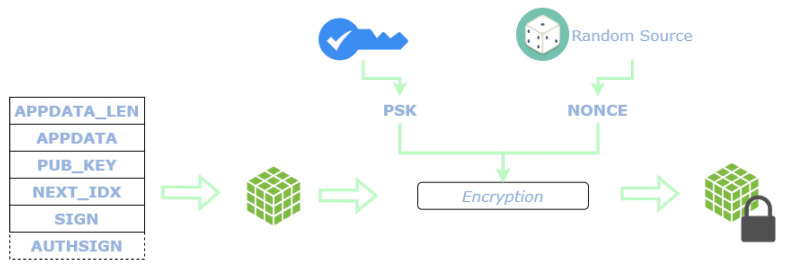
Encryption of an L2Sec message.

To preserve the confidentiality of the data, which will be public in the Tangle, every L2Sec message is encrypted as shown in Figure 7. The encryption is carried through a symmetric cryptographic key and a nonce employed as initialization vector. The encryption key is pre-shared (Pre-Shared Key - PSK) among the author of the messages and the various subscribers. The encryption is performed over all the fields of the L2Sec message with the XSalsa20 cipher [27].

This cipher protects the confidentiality by encrypting the data. According to [28], this encryption algorithm is a stream cipher which is able to reach a higher throughput than the Advanced Encryption Standard (AES) while maintaining the same security level. The encryption is backed up by the additional Poly1305 Message Authentication Code (MAC) as mechanism to verify the integrity of the message. The key employed to encrypt the data is a Pre-Shared Key (PSK) between the parties involved in the communication (i.e., author and subscribers). This work does not address how the PSK is exchanged between the parties, however the mechanism for key exchange are solid and well described in literature [29,30,31,32].

It has to be pointed out that the structure of the chain of messages together with AEAD provide *forward secrecy* (FS) of the data stream. Indeed, even if a adversary is able to discover the encryption key is still unable to recover past messages belonging to the same data stream.

After the encryption, the L2Sec message is ready to be sent and anchored to the Tangle. The final message encapsulated in a Chrysalis indexation payload is composed of the encrypted L2Sec message with the nonce employed for the encryption.

## 5. Hardware Secure Element

IoT devices, although affected from the limitations discussed in Section 2.1, are also subject to continuous technological advancements. As a matter of fact, they integrate an increasing number of peripherals. Moreover, the security domain is gaining an increasing interest. Security threats and malwares are affecting the IoT as well as Industrial-IoT (IIoT) devices. The need for cybersecurity is widespread due to increasing attacks surface and adversary capabilities [33,34]. Therefore additional hardware peripherals are often employed to provide security functionalities to custom solutions. In particular, hardware secure elements are exploited to provide additional computational capabilities in cryptographic operations or to provide additional security functionalities required by the applications [35]. They provides special storage capability that enable secure storage of sensitive data (e.g., encryption keys and other secrets). This storage can be considered secure if special protection mechanisms against physical attacks (e.g., chip decapping) are deployed. Also side-channel attacks, such as Differential Power Analysis (DPA), can be carried out to recover secrets within the security module [36,37,38], thus appropriate shielding solutions are provided by the vendors [39]. Among the other functionalities, secure elements provide a unique digital identity tied to the specific device [40].

### 5.1. STSAFE-A110

The STSAFE-A110 [14,41,42] is a highly secure solution that acts as a secure element providing authentication and secure data management services. It is a complete service solution that includes a secure operating system that operates on the latest generation of secure microcontrollers. The STSAFE-A110 is a hardware chip that can be mounted on a variety of IoT devices and provides the following functionalities:

- uniqueness of a device by means of an embedded unique serial number;
- pairing to establish a secure communication channel with symmetric cryptography between the MCU and the secure element;
- secure counters to maintain application data values;
- secure cryptographic key management stored into the secure element;
- secure storage in a configurable non-volatile memory (NVM);
- asymmetric cryptography functions employing Elliptic-Curve Cryptography (ECC) and Elliptic-Curve Digital Signature Algorithm (ECDSA) with SHA-256 or SHA-384 for digital signature generation and verification;
- secure data encryption through wrapping/unwrapping of envelopes containing secured data;
- protection mechanisms against logical and physical attacks.

Moreover, it is indeed CC EAL5+ AVA_VAN5 Common Criteria certified and DPA countermeasure licensed hence resistant to side-channel attacks. The STSAFE-A110 is integrated, among other sensors and peripherals, in the development board employed as the target device of this work The secure element is connected to the MCU through an I2C bus, reaching a transmission speed up to 400 kbps [13]. On the software level, the firmware application running on the MCU integrates the set of API [43] that allows the developer to exploit the ST-SAFE as a hardware crypto engine and as a secure facility to store sensitive information.

### 5.2. Integration with L2Sec

To harden the security of the L2Sec protocol this work exploited the security functionalities of the STSAFE-A110 by means of the SDK X-CUBE-SAFEA1, available at [43]. The set of API functions allow the protocol implmentation to directly interact with the secure element, abstracting the details of physical hardware communication.

This work enhanced the L2Sec protocol design, described in Section 4, leveraging the STSAFE-A110 functionalities described in Section 5.1 to provide (*i*) secure communication between the MCU and the STSAFE-A110, (*ii*) securely generate random numbers for security primitives, (*iii*) store securely sensitive data and, (*iv*) to provide the additional authentication signature.

Although the secure element is resilient against several physical attacks, a knowledgeable adversary with physical access to the target devices might be able to intercept the data flowing on the I2C communication bus. In order to guarantee the confidentiality of the information and commands exchanged on the physical data-bus, the setup of common shared key between the MCU and the STSAFE-A110 takes place. This operation, called *pairing* is initiated by the MCU and it has to be performed only once in the lifetime of the product. The MCU decides the cryptographic keys to secure the local communication. Considering the nature of the operation and the security implications (i.e., the transmission is in plain-text), the pairing should be performed in a secure environment, ideally at factory time. During pairing operation, the STSAFE-A110 automatically stores the host keys into its private memory slots. The MCU securely store this information as well (e.g., by storing into a free sector of the Flash memory and retrieving them at boot or when needed). The pairing enable the confidentiality and integrity of the communication, so that the STSAFE-A110 is always able to detect data tampering operations.

L2Sec requires frequent generation of random quantities for security purposes. If an adversary is able to predict or to substitute the random data generated in a way he can control, then the security of the system is compromised. Using a software Pseudo-Random Number Generator (PRNG) might yield good random numbers that ensures unpredictability. However, the values produced by a PRNG are completely predictable if the seed and generation algorithm are known. Resorting to True-RNG (TRNG) ensures a non-deterministic random number generation that depends on some unpredictable physical measures (e.g., temperature). The MCU in the target device embeds a RNG peripheral but lacks a source of entropy directly connected to it. A possible approach would be to employ the sensors (e.g., temperature, humidity, etc.) of the target device as a source of entropy, sampling the quantity and produce the random number. This way however leads to additional computations which are rendered useless by the presence of a secure element. Instead of enabling and manage an additional peripheral on the MCU, L2Sec resorts directly to the TRNG capabilities of STSAFE-A110, which is already enabled for the other functionalities and specialized for security applications. This RNG is employed for the generation of the seeds needed for the indexes and the nonce used in the encryption.

The PSK plays a very important role for maintaining the confidentiality of the data in the L2Sec protocol. Any party able to decrypt a L2Sec message is able to understand the content and breaking the confidentiality. For this reason the PSK should be kept as secure as possible. The encryption/decryption requires the PSK to be stored locally. L2Sec leverages the function of STSAFE-A110 to store the PSK on the NVM memory and retrieve it just as long as it is necessary for the encryption/decryption.

In order to avoid frequent access to the STSAFE-A110 NVM, it is possible to encrypt the PSK with a secret key stored onto the STSAFE-A110. This secret key is only known to the STSAFE-A110 (i.e., even the MCU is not able to retrieve it). This operation *wraps* the PSK into an encrypted envelope. Then, at every usage of the PSK, the enveloped is unwrapped (i.e., decrypted) by the STSAFE-A110 on-the-fly and deleted from the RAM immediately after use. In this way, the PSK can remain in an encrypted form (i.e., the *envelope*) stored in the RAM. This mechanism reduces the time the PSK in plain-text is vulnerable to snooping attacks into the RAM.

Finally, L2Sec makes use of the STSAFE-A110 for the generation/verification of the Authentication Signature to provide a means of proving and verifying the identity of the data source. As mentioned in Section 5.1, the STSAFE-A110 is able to provide and to maintain the electronic identity of the target device in the form of an asymmetric key-pair and the related public key certificate stored in the NVM. The private key is secretly stored within a key slot of the STSAFE-A110.

## 6. Testbed and Results

The L2Sec protocol defines the interactions between an IoT node and the IOTA Tangle. The experimental testbed employs a single constrained IoT device as Author and Subscriber. The experiments do not consider the interactions among different devices because they would involves the evaluation of the performances of the Tangle; that is outside the scope of the paper.

The L2Sec protocol has been implemented as C library on top of a bare-metal firmware together with the IOTA-C library [20] ported and customized to work on microcontrollers of the STM32 family. Cryptographic operations leverage the libsodium library [21]. L2Sec is freely released as open source repository on GitHub [44].

A simple sensor application has been tested on top of the L2sec protocol. The application acts as an author: it queries some of the sensors available on the board, wraps the data and anchor it to the IOTA Tangle for subscribers. The subscriber reads and verifies each message of the stream and plots the application data.

Considering that the firmware is compatible with STM32 family and the protocol can be easily ported to other IoT platforms, the test results are discussed considering the case with and without the use of the STSAFE-A110.

### 6.1. Hardware Platform

L2Sec has been developed, tested and profiled on the development-board B-L4S5I-IOT01A [13] produced and manufactured by STMicroelectronics. The board embeds the STM32L4S5VIT MCU, which is based on Arm^®^ Cortex^®^—M4 core with 2 Mbytes of Flash memory and 640 Kbytes of RAM. The board embeds an additional external memory (64-Mbit Quad-SPI), which is however not needed by L2Sec implementation. The board is equipped with the Inventek ISM43362-M3G-L4 Wi-Fi module, compliant with the Wi-Fi standards 802.11 b/g/n, and the STSAFE-A110 secure element, described in Section 5.1. The complete characteristics and the list of additional peripherals can be found in [45,46]. The sensor application on top of L2Sec leverages capacitive digital sensor for relative humidity and temperature (HTS221).

### 6.2. Timing Performances

This section provides the measurements of the time required to prepare and consume an L2Sec message through the IOTA Tangle. The data received from the application layer is encapsulated in an L2Sec message. The test payload amounts to 138 bytes. The core functions are Wrap and Unwrap, the former takes the application data and encapsulates it in an L2Sec message, by encrypting and signing it; the latter performs the specular operation. The results in Table 1 and Table 2 have been collected separately to achieve better granularity of the results and to identify computational bottleneck for further optimization. The measurements do not take into account the instructions not necessary for the operation of the protocol such as printing on serial or led blinking.

Table 1 reports the execution time of the functions to produce an L2Sec message without the use of STSAFE-A110.

Every operation is performed on the MCU. The PSK for encryption and decryption is used straightly because it is stored in plain-text form. The cipher implementation provided in libsodium does not require lookup operations and avoid the possibility of timing attacks. The most computational intensive operation is the signature in either the author (Sign-Gen.) and the subscriber (Sign-Verif.). In this case, only the SIGN signature is embedded in the L2Sec message.

Table 2 provides the time required to prepare and consume an L2Sec message leveraging the STSAFE-A110.

When the STSAFE-A110 is enabled, both the signatures for integrity and for authentication are embedded in the message, see Figure 2. The subscriber verifies both as well. The encryption and decryption functionalities employ the PSK in its wrapped form, thus the PSK has to be unwrapped and cleared every time it is employed, leading to longer execution time. The Authentication Signature generation (AuthSign-Gen.) includes the time to compute the HMAC-SHA256 of all fields, while the verification (AuthSign-Verif.) requires to extract and verify the public key of the STSAFE-A110.

As expected, the timing performances are lower with the hardware secure element. This is due to additional operations to be performed that concern the Authentication Signature and the PSK. Such operations lead to an additional communication overhead between the MCU and the STSAFE-A110. Although the time measurements are greater, the security improvement is also significant. Moreover, several optimizations are possible. For instance, during a data stream transmission, the PSK can be unwrapped only once and at the end of the stream can be reset. Also, the public key for the verification of the authentication signature can be extracted only once and stored in plain text on the MCU. Such simple optimizations would lead to a considerable improvement in performance. Conversely, a pure software implementation is much faster, but less secure as well.

Timing results for sending and receiving data to and from the IOTA Tangle are not provided. This is due to the fact that the communication with the IOTA Tangle depends on connection speed, network congestion and transactions validation. All these parameters cannot be directly controlled by L2Sec and are outside the scope of this paper.

### 6.3. Memory Consumption

The total size of a L2Sec message is 332 bytes and 268 bytes, respectively in the case with and without the Authentication Signature field, see Figure 2. The overhead derived from the header fields amounts to 128 bytes without considering the Authentication Signature and 192 bytes taking it into account. Table 3 summarizes the type and the data size of the fields of the L2Sec message, showing for each the impact as a percentage of the total size with and without the use of the STSAFE-A110.

The major contribution derives from the Application Data field, followed by the two signatures. In both cases the Application Data field, together with its length is kept fixed at 138 bytes. The total size of the message after the encryption is 308 bytes, while including the Authentication Signature amounts to 372 bytes (24 bytes of which, are employed for the nonce).

This firmware is built with GNU arm-gcc compiler. With respect to the target device, the memory footprint (i.e., the size of the binary) of the whole X-CUBE-IOTA1 package firmware employing the hardware secure element is considered. The static analyzer reports 751.93 KBytes (36.72%) of used flash memory out of the total of 2 MBytes available. It has to be pointed out that the resulting binary image is composed of all the functionalities of the package, an external pre-compiled library for encryption functionalities (i.e., libsodium) and embedding hard-coded constants, such as the data of the menu to be printed. The latter is included in the experimental results for the sake of completeness, it directly contributes to the total code size, but it can also be discarded from the computation because not strictly needed. The memory also stores X.509 certificates and network configurations; both affect the size of the memory used. Moreover, no special settings concerning the code size are given to the compiler. Several additional optimizations can be employed to reduce the total memory footprint in a production environment.

More in detail, the memory size of the core functions of L2Sec are reported in Table 4. Also these results are obtained by resorting to the static analysis of the binary application. The modularity of the implementation permit to embed only a portion of the code corresponding to either the author of the data or the subscriber. The experimental results consider the code memory used by both roles.

The functions containing the logic of the protocol (i.e., send* and receive*), are among the largest. Also the routines used to prepare the L2Sec data, (i.e., l2sec_wrap and l2sec_unwrap) shows a considerable code size. The difference in the code size of the L2Sec core functions with and without the use of STSAFE-A110 is negligible (roughly to 376 bytes). However, without the STSAFE-A110 the total code size decreases. The version without the library for the hardware secure element functionalities, instead, amounts to 736.91 KBytes resulting in 35.98% of flash usage. Table 4 reports also the size cost of additional functions calls to external libraries and module (i.e., crypto*, iota*). The IOTA-C library embeds a module for buffer allocation and management employing dynamic memory; the X-CUBE-IOTA1 package inherits from it. Although L2Sec is written on top of IOTA-C, it does not make use of dynamic memory allocation. The total memory occupied is 138.60 KB out of 640 KB (21.66%), while it drops to 137.81 KB (21.53%) disabling the STSAFE-A110. Both values provide the measurement of local variables and static data structures of the L2Sec functions.

### 6.4. Power Consumption

In order to provide a complete evaluation of the L2Sec performances the test comprised some power consumption measurements. The development board has been powered on and left in idle state for 24 h. In this state, the device is turned on but it does not execute any task, nor its sensors and its peripherals receive power. The power consumption in idle state is 0.84 Wh. For comparison, the measure is repeated with the device in its operational state. In this setting, the device executes continuously the same tasks, i.e., it transmits a continuous stream of data over the IOTA Tangle. In this case the power consumption of the board amounts to 1.25 Wh. Apart from the MCU and the STSAFE-A110, the other peripherals enabled and affecting actively the consumption are the barometric sensor and the WiFi Module. To provide additional granularity of the power consumption measurements requires instrumentation with greater accuracy. Nevertheless, the measurements here provided are an estimate of the power consumption in the worst-case, where the device and its peripherals are always active. In a real-case scenario the software controller logic would be able to to reduce the consumption resorting to low-power sleep states of the peripherals not needed.

## 7. Conclusions and Future Work

This paper has presented the design and the implementation of L2Sec, a cryptographic protocol to securely exchange data over the IOTA Tangle suitable for constrained IoT devices. To the best of the author’s knowledge, today there is not another viable solution for MCU-based IoT devices. The profiling activities of this first implementation has shown the effectiveness of the protocol and suggests that L2Sec is suitable to be ported on other limited IoT platforms. The experimental results have addressed the performances of L2Sec with and without the adoption of an hardware secure element. The adoption of such cryptographic hardware increases the robustness of the protocol. Future works will be devoted to further analysis of the protocol in IoT constrained field and its integration with a suitable Trusted Execution Environment (TEE) technology. The underlying idea is to take advantage of a proper combination of TEE and hardware security modules to make it more robust to adversaries while not degrading performances.

## Figures and Tables

**Table 1:** Execution time of L2Sec functionalities on STM32L4S5VIT MCU with STSAFE-A110 disabled. Time is expressed in milliseconds (ms).

Encryption	Decryption	Sign-Gen.	Sign-Verif.	Wrap	Unwrap
2	1	63	2	185	3

**Table 2:** Execution time of L2Sec functionalities on STM32L4S5VIT MCU with STSAFE-A110 enabled. Time is expressed in milliseconds (ms).

Encryption	Decryption	AuthSign-Gen.	AuthSign-Verif.	Wrap	Unwrap
1012	993	172	570	1388	1566

**Table 3:** Detailed size of the fields embedded in a L2Sec message.

Field	Type	Size (bytes)	Percentage % (No Auth. Sign.)	Percentage % (with Auth. Sign.)
Data Length	byte	2	0.75	0.60
Application Data	byte	138	51.49	41.56
Public Key	byte	32	11.94	9.64
Next Index	byte	32	11.94	9.64
Signature	byte	64	23.88	19.28
Auth. Signature	byte	64	-	19.28

**Table 4:** Code size of significant functions for L2Sec implementation. All sizes are expressed in bytes.

Function Name	Size (STSAFE OFF)	Size (STSAFE ON)	Variation
send_l2sec_protected_stream	332	332	0
send_l2sec_protected_msg	172	172	0
l2sec_wrap	644	792	148
send_l1_message	544	544	0
receive_l2sec_protected_stream	148	148	0
receive_l2sec_protected_msg_by_index	1014	1014	0
receive_l2sec_protected_msg_by_id	96	96	0
get_l1_message_by_id	236	236	0
l2sec_unwrap	660	888	228
iota_blake2b_sum	54	54	0
iota_crypto_sign	66	66	0
crypto_secretbox_easy	78	78	0
crypto_secretbox_open_easy	80	80	0
crypto_sign_ed25519_verify_detached	28	28	0

## Data Availability

Not applicable.
